# Brain microischemic phenomena in a woman receiving bevacizumab treatment: a case report

**DOI:** 10.1186/1752-1947-5-84

**Published:** 2011-02-27

**Authors:** Carlo C Quattrocchi, Andrea M Alexandre, Giuseppe Tonini, Yuri Errante, Rosario F Grasso, Bruno Beomonte Zobel

**Affiliations:** 1 Interdisciplinary Center for Biomedical Research, Department of Radiology, University Campus Bio-Medico of Rome, via Longoni, 47 I-00155 Rome, Italy; 2 Department of Bio-imaging and Radiological Sciences, Catholic University of Sacred Heart, Policlinico A. Gemelli. Largo F. Vito, I-00100 Rome, Italy; 3 Interdisciplinary Center for Biomedical Research, Oncology, University Campus Bio-Medico of Rome, via Longoni, 47 I-00155 Rome, Italy

## Abstract

**Introduction:**

Several adverse events have been associated with the use of bevacizumab during the treatment of neoplasms such as colorectal cancer, breast cancer, non-small cell lung cancer, pancreatic cancer and renal cell carcinoma. The present case demonstrates how focal neurological symptoms lead to the magnetic resonance imaging-based differential diagnosis between focal parenchymal metastases and microischemic phenomena, with crucial implications for patient management.

**Case presentation:**

We describe the case of a 37-year-old Italian Caucasian woman with metastatic colon cancer who developed focal neurological symptoms during a chemotherapy regimen involving the use of bevacizumab. Brain magnetic resonance imaging examination revealed millimetric lesions with restricted diffusion without perilesional edema or contrast enhancement after gadodiamide intravenous injection, suggestive of acute microischemic phenomena. This complication is very rare but clinically significant.

**Conclusion:**

The differential diagnosis in patients with cancer undergoing bevacizumab treatment should include microischemic phenomena.

## Introduction

We describe the case of a patient with metastatic colon cancer who developed focal neurological symptoms during a chemotherapy regimen including bevacizumab. Some millimetric lesions were detected by a first magnetic resonance imaging (MRI) examination and were not detectable on the MRI examination performed six months later. In patients with cancer undergoing bevacizumab treatment, the occurrence of neurological focal symptoms leads to an MRI differential diagnosis between focal parenchymal metastases and microischemic phenomena, with crucial decisions that must be made for patient management.

## Case presentation

A 37-year-old Italian Caucasian woman underwent a colonscopy that revealed a polypoid formation 28cm from the external anal margin. The biopsy showed areas of adenocarcinoma in the context of tubulovillous and villous adenoma with mild to severe dysplasia. Computed tomography (CT) staging was negative for regional or distant metastases. Surgical removal was performed by partial colectomy. The tumor histology confirmed the diagnosis of adenocarcinoma with infiltration of the serosa and pathological TNM staging of pT4pN1M0.

A follow-up CT examination three months later revealed eight focal hepatic lesions distributed throughout both lobes. Chemotherapy treatment with the folinic acid, fluorouracil and oxaliplatin (FOLFOX) scheme was started, and the patient showed a partial response after the fourth course of treatment. She underwent surgical resection of metastases localized at hepatic segments IV and V. CT examination showed disease progression in the lung and liver parenchyma six months later. Several lines of treatment were started, including XELOX (capecitabine plus oxaliplatin), FOLFIRI (folinic acid, fluorouracil and irinotecan) and radiofrequency thermoablation, with no response. CT showed a partial hepatic response after 12 courses of cetuximab and irinotecan therapy, but hepatic progression was observed after 24 courses. Therefore, chemoimmunotherapy with bevacizumab (Avastin; Genentech, South San Francisco, CA, USA) and FOLFOX was started, but it was suspended after nine cycles as the patient developed left hemiparesis, hemifacial left anesthesia and right-hand paresthesia.

**Figure 1:**
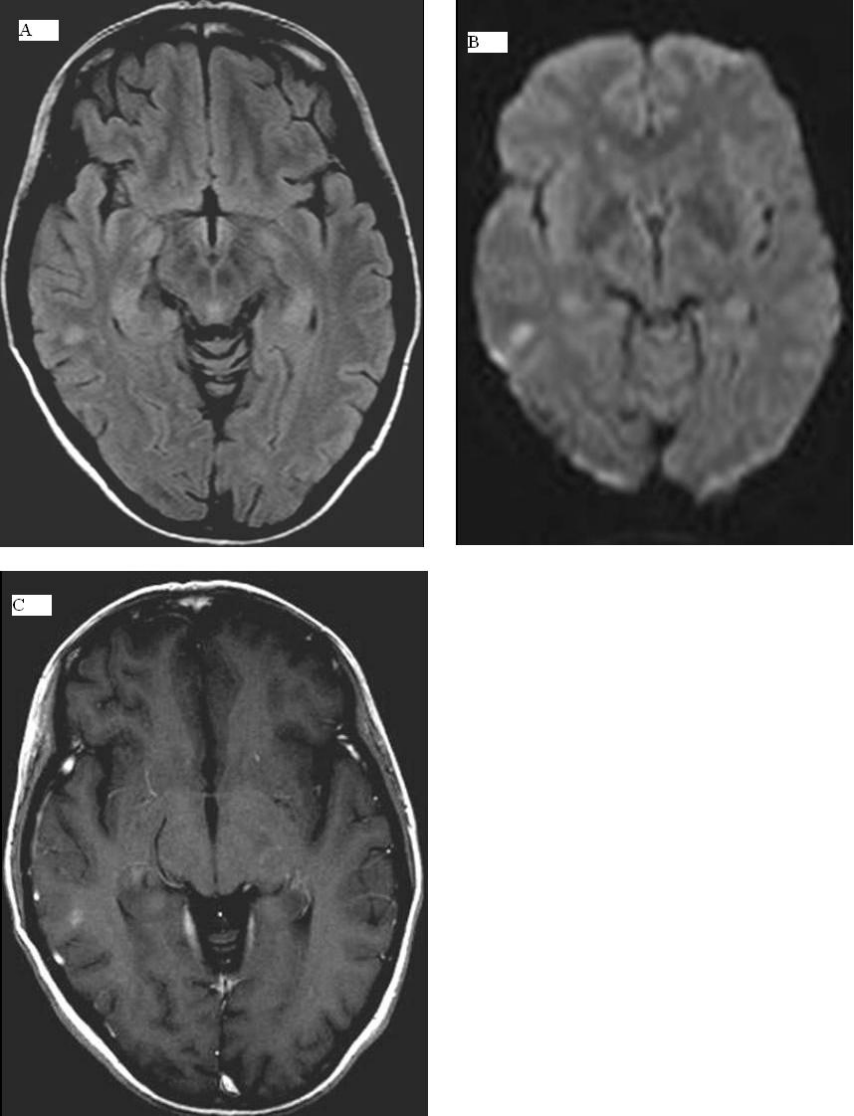
**(A) Right temporooccipital lesion can be easily detected as a hyperintense spot in T1-weighted sequences **. **(B) **The lesion shows restricted diffusion, an absence of perilesional edema. **(C) **No enhancement is observed after gadodiamide injection. These findings are suggestive of areas of microischemic phenomena.

**Figure 2:**
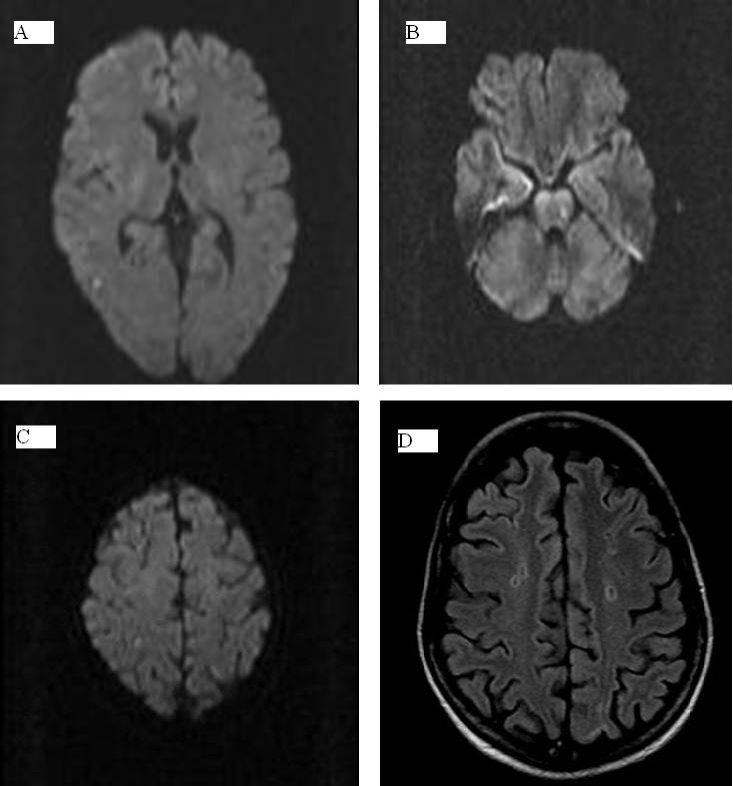
**Other lesions suggestive of areas of acute embolic strokes are **(A) **the right parietal lobe, **(B) **the left pontine region and **(C) **the right parietal lobe (alternate view) **. **(D) **Another bilateral centrum semiovale lesion not detected on diffusion-weighted images, without contrast enhancement, is hypointense in this fluid attenuated inversion recovery (FLAIR) image with a hyperintense gliotic peripheral ring, suggestive of small vascular ischemic microlacunae.

**Figure 3:**
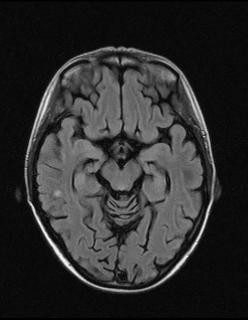
**FLAIR image obtained at the patient's one-year follow-up magnetic resonance imaging (MRI) examination demonstrating the subcortical lesion at the level of the right temporooccipital white matter **. The lesion was unchanged with regard to size and signal intensity compared with the previous MRI (see Figure 1A).

A brain MRI scan showed three millimetric lesions located in the right temporooccipital lobe (Figure 1A), the left pontine region (Figure 2B) and the right parietal lobe (Figure 2C) with restricted diffusion (Figures 1A to 1C) and no enhancement after gadodiamide injection. These findings, that is, hyperintensity on fluid attenuated inversion recovery (FLAIR) images, slight enhancement on postgadolinium T1-weighted images, restricted diffusion and no contrast enhancement, were suggestive of areas of acute microischemic strokes. Although unusual in the context of stroke, the subcortical lesion at the level of the right temporooccipital white matter was confirmed to be unchanged with regard to size and signal intensity on FLAIR images obtained at the one-year follow-up examination (Figure 3). Moreover, other bilateral centrum semiovale lesions not detected on diffusion-weighted images and not showing contrast enhancement were hypointense with a hyperintense gliotic peripheral ring visualized on FLAIR images, suggestive of areas of chronic microischemic origin (Figure 2D). These lesions appeared as millimetric spots of hyperintensity on T2-weighted images obtained at the one-year follow-up examination. No other risk factors for thromboembolic events were recognized: the patient's clinical history was negative for hypertension, hypercholesterolemia, hypertriglyceridemia, diabetes, obesity, smoking, atrial fibrillation, heart disease, atrial or ventricular septal defects and previous episodes of thrombosis or symptoms correlated to thrombosis. Her pharmacological history was negative for anticoagulant or procoagulant drugs. Her platelets were 161 × 10^3^/μL (normal range, 150 to 450), her International Normalized Ratio was 1.08 (normal range, 0.85 to 1.16) and her activated partial thromboplastin time ratio was 0.84 (normal range, 0.82 to 1.20).

An ultrasound Doppler study was obtained, which showed normal morphology of supraaortic vessels. In addition, the patient's electrocardiogram and echocardiogram were normal.

Low-molecular-weight heparin (6,000 IU twice daily), edema therapy (8 mg of dexamethasone twice daily) and antiplatelet therapy (200 mg/day aspirin) were administered, resulting in complete resolution of the patient's neurological symptoms.

## Discussion

Bevacizumab, a humanized antibody directed against the vascular endothelial growth factor (VEGF) that is used as an angiogenesis inhibitor, has been examined in combination with chemotherapeutic agents in several clinical trials in patients with advanced colorectal cancer [1], even as a first-line treatment [2]. The addition of bevacizumab increased the overall response rate and extended median survival. In the past four years, bevacizumab has been used with increasing frequency for the treatment of other neoplasms, such as breast cancer, non-small cell lung cancer, pancreatic cancer and renal cell carcinoma.

Several adverse events have been associated with the use of bevacizumab: hypertension (the most common side effect), gastrointestinal perforation, wound-healing complications, hemorrhage, arterial thomboembolic events, proteinuria, congestive heart failure, leukopenia and diarrhea [3]. Arterial thromboembolic events have been observed in 0.9% of the cases in the BEATrial [3] and in 2.1% of the cases in the BRiTE Registry [4]. The mechanism of concurrent thrombosis and bleeding during bevacizumab treatment is not clear, being related to the role of VEGF in maintaining a healthy endothelium.

## Conclusion

Vascular events involving the central nervous system have been reported as reversible posterior leukoencephalopathy syndrome following a bevacizumab or FOLFIRI treatment regimen for metastatic colon cancer, which are likely related to high systolic blood pressure levels [5]. Furthermore, as thromboembolic events and microischemic phenomena are a well-known complication of bevacizumab chemotherapeutic treatment [6], the occurrence of neurological focal symptoms leads to an MRI-based differential diagnosis between focal parenchymal metastases and microischemic phenomena, which lead to crucial decisions for patient management.

## Consent

Written informed consent was obtained from the patient for publication of this case report and accompanying images. A copy of the written consent is available for review by the Editor-in-Chief of this journal.

## Competing interests

The authors declare that they have no competing interests.

## Authors' contributions

GT and YE analyzed and interpreted the patient data regarding the primary disease (colon cancer) and decided on the therapeutic strategy. CCQ, RFG and BBZ performed the brain magnetic resonance imaging. CCQ and AMA were the major contributors in writing the manuscript. All authors read and approved the final manuscript.
